# The paradox of plastic bag legislation: How bans and taxes affect PM2.5 air pollution in 208 countries

**DOI:** 10.1016/j.heliyon.2024.e40641

**Published:** 2024-11-22

**Authors:** Rafi Amir-ud-Din, Muhammad Khan, Rao Muhammad Atif, Saliha Khalid

**Affiliations:** Department of Economics, COMSATS University Islamabad, Lahore Campus, Lahore, 54000, Pakistan

**Keywords:** PM2.5, Plastic bag ban & taxation, Environmental policy impact, WHO air quality guidelines

## Abstract

Widespread use of plastic bags contributes to elevated air pollution levels worldwide, prompting various regulatory measures such as bans and taxes aimed at reducing plastic pollution. The objective of this study was to analyze the impact of these plastic bag bans and taxes on PM2.5 air quality across 208 countries from 1960 to 2021, using Fixed Effects, Driscoll and Kraay, and GMM models. Results indicate that bans generally reduce the population's exposure to PM2.5 above WHO guidelines, but increase exposure above the Interim Target-1, while reducing it above Interim Target-3 in some models. Conversely, taxes on plastic bags significantly increase both mean annual PM2.5 exposure and the proportion of the population exposed to levels surpassing all WHO targets. The combined effect shows a decrease in exposure due to bans, except for an increase above Interim Target-3, while taxes increase exposure across all measures. These findings highlight complex interactions between plastic bag policies and air pollution, emphasizing the need for careful policy design. While plastic bag bans effectively reduce PM2.5 exposure, taxes on plastic bags unexpectedly increase it, emphasizing the need for carefully designed policies to prevent unintended increases in air pollution.

## Highlights

• Bans reduce PM2.5 exposure but raise levels above IT-1.
• Taxes on bags increase PM2.5 across WHO targets.
• Study spans 208 countries, 1960–2021 data.
• Complex ties: plastic bag policies and air pollution.
• Policy design crucial for effective PM2.5 reduction.

## 1 Introduction

The production of plastic has reached staggering levels, with over 300 million tons manufactured annually worldwide, a figure that is nearly equivalent to the weight of the entire human population [1]. This immense production contributes to the approximately 380 million metric tons of plastic generated each year [2].

The scale of this crisis extends to the oceans, the final resting place for more than 10 million tons of plastic every year [1]. This relentless disposal into the marine ecosystem has made plastic waste a dominant constituent of ocean pollution. The situation is so dire that if left unchecked, the amount of plastic in the oceans could surpass the total weight of all the fish by the year 2050, a sobering projection that underscores the magnitude of this crisis [3].

A significant contributor to this escalating problem is the ubiquitous single-use plastic bag. With a staggering production rate of five trillion such bags annually around the globe, their role in exacerbating plastic pollution cannot be overstated [2]. Predominantly designed for single-use, these plastic bags are a major source of the plastic waste that ends up in landfills and oceans, accounting for a substantial portion of the 79 % of global plastic waste not recycled or incinerated [3].

Made of non-biodegradable materials, plastic bags can take hundreds of years to decompose, thereby polluting the environment for extended periods [4]. Furthermore, the degradation of these bags creates macro and microplastics that worsen land and ocean pollution, posing serious hazards to public health and animals [5]. Additionally, microplastics are ubiquitous in the natural environment and pose significant risks to global health by causing acute, chronic, and genotoxic damage in living organisms [6]. Urban areas are particularly vulnerable to plastic pollution because plastic bags can choke water channels and cause urban flooding [[7], [8], [9]]. Despite attempts to find alternative uses for plastic bags through upcycling and promoting environmental awareness, recycling strategies, and behavior change communication (BCC), the challenges of waste management and the limited capacity of governments in developing countries to manage their waste effectively remain significant hurdles [7,[9], [10], [11], [12], [13]].

The plastic pollution crisis, especially concerning the production of five trillion plastic bags per year worldwide, has prompted various countries to implement taxation and bans on single-use plastic bags. These measures' effectiveness varies significantly based on factors like public awareness, attitudes, and law enforcement capacity. For instance, a ban in Kenya significantly reduced plastic pollution, while law enforcement challenges in Nepal and Indonesia led to increased use of plastic bags [[14], [15], [16], [17]].

Moreover, the disposal of non-biodegradable plastic bags, which take hundreds of years to decompose, has become an environmental hazard. Waste management of these bags is challenging due to inadequate infrastructure and policies in most governments, leading to attempts at alternative uses like upcycling designs [4,11,12].

Several countries have taken regulatory measures, such as bans and taxes, to reduce the usage of single-use plastic bags. However, the success of these measures is context-specific, and in some cases, they may create underground markets or normalize the violation of regulatory measures. Taxes can also disproportionately affect lower-income households [[18], [19], [20], [21], [22]].

Countries like Rwanda, Kenya, Eritrea, China, and Uganda have implemented bans on single-use plastic bags to enhance their environmental profiles. Yet, enforcing these bans can be challenging due to industry resistance or lack of sustainable waste management planning. For effectiveness, strong regulatory frameworks and enforcement mechanisms are essential [9,16,[23], [24], [25]].

To address these challenges, this study aims to analyze the impact of plastic bag bans and taxation on PM2.5 air quality indicators across 208 countries from 1960 to 2021, assess the effect of plastic bag taxation within the same global context and timeframe, and compare the differential impacts of bans and taxes on PM2.5 exposure levels, particularly focusing on population exposure above WHO guidelines and interim targets. The rationale behind this research stems from the critical need to understand the broader environmental implications of plastic bag regulatory measures beyond their immediate effects on plastic pollution. By addressing the following research questions—how do plastic bag bans affect PM2.5 air quality indicators globally? What is the effect of plastic bag taxation on PM2.5 air quality indicators worldwide? and do plastic bag bans and taxes have differential impacts on PM2.5 exposure levels—this study seeks to provide comprehensive empirical evidence on the effectiveness of plastic bag policies in improving air quality. The potential contribution of this study lies in offering valuable insights for policymakers to design more effective environmental regulations that mitigate air pollution while addressing plastic waste management.

## 2 Literature review

### 2.1 Plastic and environment quality: some causal mechanisms

To explore the causal mechanism through which plastics contribute to air pollution, it's essential to understand the various processes involved in the degradation of plastics and their impact on the environment.

The environmental impact of plastic usage is particularly profound in the Anthropocene era. Common plastics, typically made from synthetic organic polymers like polyethylene, polypropylene, and polystyrene, have extensive lifecycles. They break down into smaller fragments - mesoplastics, microplastics, and nanoplastics - which can disperse in the atmosphere, travel vast distances, and contribute to a cycle of plastic transport. These smaller plastic pieces can carry heavy metals and polycyclic aromatic hydrocarbons, adding to air pollution [26].

The process that converts plastics into smaller micro and nanoplastics is complex. Degradation occurs through hydrolysis, oxidation, photodegradation, mechanical corrosion, and biological degradation. In photodegradation, exposure to ultraviolet light creates additional oxygen functional groups on the plastic surface, rendering it more brittle and susceptible to chemical degradation. This degradation can result in various reactive oxygen species, potentially exacerbating photo ageing effects. Studies suggest that when animals ingest plastic bags, the microplastics can be further broken down into smaller pieces, which are then reintroduced into the environment through animal defecation [27]. Furthermore, environmental factors such as local meteorological conditions and trace element concentrations, as observed in Tablas de Daimiel National Park, may significantly influence the degradation and distribution of microplastics in natural ecosystems [28].

The degradation of mismanaged macro plastic waste leads to the formation of MPs and NPs, which are reported in various environmental compartments, including urban air. These particles can absorb toxic organic pollutants like polycyclic aromatic hydrocarbons (PAHs). Factors such as specific surface area, particle size, crystallinity, and the polymer's spatial arrangement influence this absorption. Transformation mechanisms like photooxidation and abrasion further alter the microplastics' mechanical integrity and chemical behavior [29]. Further fragmentation of primary and secondary MPs results in the creation of nanoplastics (NPs) [30].

Microplastics (MPs) contribute to air pollution through a complex interplay of environmental processes and human activities. Atmospheric deposition is a key factor, where microfibers and other MPs are introduced into the environment, particularly in urban areas with high air pollution levels. Fragmentation and weathering of larger plastic items break down into smaller particles under various environmental conditions, including UV radiation and physical stress. These weathered and fragmented MPs can become airborne, adding to air pollution. Human activities such as commercial and industrial operations, the use of synthetic clothing, and improper waste management practices are significant contributors to the release of MPs into the environment [31].

The causal mechanism linking plastics to PM2.5 pollution is primarily through the formation and degradation of secondary microplastics. These microplastics originate from the fragmentation of larger plastic items under environmental conditions such as sunlight, heat, and physical abrasion. As plastics degrade, they break down into increasingly smaller particles, some of which fall into the PM2.5 size range. These fine particles are concerning for air quality and human health, as their small size allows them to penetrate deep into the lungs and, in the case of ultrafine particles, even enter the bloodstream, leading to systemic health effects. The impact of these microplastics on air quality is significant; even small increases in PM2.5 due to microplastics can have substantial health and economic costs [32].

Plastics, particularly polyethylene terephthalate (PET) microplastics and nanoplastics, contribute to air pollution through their ability to adsorb primary air pollutants. These particles selectively bind pollutants like CO, CO2, NO, and SO2. The adsorption mechanism is driven by a combination of electrostatic and dispersion forces, with the type of adsorption (inner or outer) dependent on the pollutant's molecular characteristics. PET microplastics don't significantly adsorb H2O and O2 but can enhance the adsorption of other pollutants, especially under the influence of ozone. This capability of PET particles to transport air pollutants contributes to air pollution across various regions [33].

### 2.2 Plastic bags and PM2.5

Plastic bags significantly contribute to environmental pollution, including PM2.5 levels, through various stages of their lifecycle: production, usage, and disposal. The production of plastic bags involves the extraction and processing of petroleum or natural gas. This stage can lead to the release of various pollutants, including PM2.5, with the extent of emissions being influenced by the specific production processes and pollution control measures in place. The link between the production of plastic bags and PM2.5 pollution is an important aspect of their environmental impact [34]. During their use, plastic bags have been identified to cause environmental issues such as soil and water pollution, impact on wildlife, and influence on vegetation biomass of soft-bottom communities [35,36]. However, their direct contribution to PM2.5 pollution in this phase is comparatively lesser.

Inappropriate disposal of plastic bags can be a major factor in PM 2.5 pollution. Open-air burning of plastic waste, common in many developing countries, releases various pollutants, most notably PM 2.5 [[37], [38], [39], [40]]. Similarly, the degradation of plastic bags into microplastics potentially contributes to PM 2.5 pollution akin to combustion emissions [41,42].

When introduced into waste management and recycling systems, plastic bags can cause operational challenges, such as machinery entanglement and complications in sorting. These disruptions could increase the overall energy use and emissions, including PM2.5, within these systems. Therefore, the pollution potential from the management of plastic waste, including PM2.5 emissions, is a crucial consideration in developing comprehensive plastic waste management strategies [34].

### 2.3 Regulation and plastic bag use

Regulatory measures have a significant impact on the use of plastic bags, as evidenced by various studies from different countries. The effectiveness of a 15 cent duty on plastic bags in Ireland, introduced in mid-2002, led to a significant reduction in plastic bag litter, dropping from 5 % of Ireland's total litter to just 0.25 % in 2003 and 2004. This reduction was attributed to the tax's acceptability and a comprehensive marketing awareness campaign [43]. Similarly, the implementation of charges on plastic bags in Botswana resulted in a sustained decrease in usage, contrasting with results in South Africa, where the continuous high prices for plastic bags were a major indicator of success [44]. In Italy, the introduction of a 5 cent tax on the production of plastic bags in 1989 made them more expensive due to the Value-Added Tax (VAT), reducing demand significantly. In Denmark, a similar tax targeted larger packaging companies, leading to a decrease in demand by 66 %, compared to 90 % in Ireland [45].

However, the effectiveness of such regulatory measures has varied. The prohibition of plastic bags in Delhi's semi-organised sector initially reduced plastic bag consumption, but the impact weakened over time due to ineffective enforcement [46]. Similarly, a ban in Islamabad Capital Territory on single-use plastic bags led to a 77.5 % decrease in their use, though smaller retailers often flouted the law [47]. Conversely, the plastic bag ban in Argentina demonstrated mixed outcomes when retailers charged consumers for their use, resulting in varied behavioral changes [48,49]. In Southern Australia and Brazil, bans encouraged consumers to opt for their own or reusable bags, enhancing environmental friendliness [50,51]. However, ineffective implementation in places like the Philippines and the Netherlands limited the bans' efficacy [52,53]. This evidence suggests that regulatory measures can effectively reduce plastic bag usage, contingent on factors like implementation strategies and public perception.

The literature reveals that although the vast production of plastics, particularly single-use plastic bags, significantly contributes to environmental pollution and PM2.5 levels [[1], [2], [3], [4], [5]], the effectiveness of regulatory measures like bans and taxes in mitigating this impact varies greatly across different contexts [[14], [15], [16], [17], [18], [19], [20], [21], [22], [23], [24], [25], [26], [27], [28], [29], [30], [31], [32], [33], [34], [35], [36], [37], [38], [39], [40], [41], [42], [43], [44], [45], [46], [47], [48], [49], [50], [51], [52]]. Successful bans in countries like Kenya led to substantial reductions in plastic pollution due to effective enforcement and public compliance [14], whereas in Nepal and Indonesia, enforcement challenges resulted in increased usage despite regulations, highlighting the critical role of law enforcement capacity [[15], [16], [17]]. Taxes have been effective in some countries, such as Ireland and Botswana, where they significantly reduced plastic bag usage [42,43], but they can also disproportionately affect lower-income households and potentially lead to unintended consequences like underground markets or normalization of regulatory violations [[18], [19], [20], [21],^21^,^22^,^22^]. Additionally, while plastics degrade into microplastics and nanoplastics that exacerbate PM2.5 pollution through processes like atmospheric dispersion and absorption of toxic pollutants [[26], [27], [28], [29], [30], [31], [32]], the entire lifecycle of plastic bags—from production, which emits pollutants depending on processes and control measures [33], to disposal practices like open-air burning contributing to PM2.5 emissions [[36], [37], [38], [39]]—impacts air quality. This comparison underscores that the success of regulatory interventions is highly dependent on implementation strategies, enforcement mechanisms, public perception, and economic considerations, emphasizing the need for comprehensive, context-specific approaches to effectively reduce plastic bag usage and improve environmental outcomes.

While there is substantial evidence on the impact of regulatory measures like bans and taxes on single-use plastic bag usage and their effectiveness in reducing plastic pollution, there is a significant knowledge gap regarding their effect on air quality, specifically on PM2.5 pollution levels. **To address this gap, this study empirically analyzes the influence of plastic bag bans and taxation on PM2.5 air pollution in a cross-country context over a substantial period. The study hypothesizes that plastic bag bans significantly reduce PM2.5 exposure levels above WHO guidelines and interim targets, that plastic bag taxation leads to a reduction in PM2.5 exposure levels, and that plastic bag bans and taxes have differential effects on PM2.5 exposure levels, with taxes potentially leading to unintended increases in pollution.**

## 3 Methods

In this study, we aim to empirically assess the impact of plastic bag bans and taxation policies on environmental quality, specifically focusing on PM2.5 air pollution levels across 208 countries from 1960 to 2021. To achieve this, we employ panel data estimation techniques, which allow us to control for both cross-sectional and temporal variations in the data.

The empirical models are specified as follows:

$$\begin{array}{c} {PM2.5}_{i,t}^{\gamma }=\beta_{0}+\beta_{1}Ban_{i,t}+\xi X_{i,t}+\epsilon_{i,t}\;\left(Eq.\;1\right) \end{array}$$

$$\begin{array}{c} {PM2.5}_{i,t}^{\gamma }=\beta_{0}+\beta_{1}{Tax}_{i,t}+\xi X_{i,t}+\epsilon_{i,t}\;\left(Eq.\;2\right) \end{array}$$

$$\begin{array}{c} {PM2.5}_{i,t}^{\gamma }=\beta_{0}+\beta_{1}Ban_{i,t}+\beta_{2}{Tax}_{i,t}+\xi X_{i,t}+\epsilon_{i,t}\;\left(Eq.\;3\right) \end{array}$$

${\text{PM}2.5}_{i,t}^{\gamma }$ represents the different PM2.5 air pollution indicators for country *i* at time *t*. The superscript γ indicates the five different PM2.5 indicators used in the analysis (as detailed in Table 1). $Ban_{i,t}$ is a binary variable equal to 1 if a country has implemented a ban on plastic bags in year *t*, and 0 otherwise. $Tax_{i,t}$ is a binary variable equal to 1 if a country has implemented a tax on plastic bags in year *t*, and 0 otherwise. $X_{i,t}$ is a vector of control variables (covariates) that may affect PM2.5 levels.

**Table 1:** Variable description.

Variable	Description	Data source
**Outcome**		
PM2.5 (mcg)	PM2.5 air pollution, mean annual exposure (micrograms per cubic meter μg/m³)	World Bank
PM2.5 (% exposed) T-1	PM2.5 pollution, population exposed to levels exceeding WHO Interim Target-1 value (35 μg/m³)	World Bank
PM2.5 (% exposed) T-2	PM2.5 pollution, population exposed to levels exceeding WHO Interim Target-2 value (25 μg/m³)	World Bank
PM2.5 (% exposed) T-3	PM2.5 pollution, population exposed to levels exceeding WHO Interim Target-3 value (15 μg/m³)	World Bank
PM2.5 (% exposed)	PM2.5 air pollution, population exposed to levels exceeding WHO guideline value	World Bank
**Covariates**		
Energy use	Energy intensity level of primary energy (MJ/$2011 PPP GDP)	World Bank
FDI (% of GDP)	Foreign direct investment, net inflows (% of GDP)	World Bank
Forest cover	Forest area (% of land area)	World Bank
Fossil fuel (%)	Fossil fuel energy consumption (% of total)	World Bank
CO2 emissions	CO2 emissions (metric tons per capita)	World Bank
GDP per capita	GDP per capita, PPP (constant 2017 international $)	World Bank
Population density	Population density (people per sq. km of land area)	World Bank
Urban population (%)	Urban population (% of total population)	World Bank
Electricity production	Electricity production from oil, gas and coal sources (% of total)	World Bank
GDP (energy use)	GDP per unit of energy use (constant 2017 PPP $ per kg of oil equivalent)	World Bank

“PM2.5 air pollution, mean annual exposure (micrograms per cubic meter)" provides the average level of PM2.5 pollution experienced in a specific area over a year, encompassing all individuals regardless of their exposure exceeding specific targets (Table 1) [54]. It offers an overall measure of pollution levels in a given location. The three target variables PM2.5 pollution, population exposed to levels exceeding WHO Interim Target-1, Target-2, and Target-3 value focus on the portion of the population exposed to PM2.5 pollution levels surpassing the interim targets set by the World Health Organization [54]. It may be noted that the target values associated with Target-1, Target-2, and Target-3 are 35 μg per cubic meter (μg/m³), 25 μg per cubic meter (μg/m³), and 15 μg per cubic meter (μg/m³) as an annual mean concentration, respectively. Target-3 is the most stringent air quality standard among the three. Finally, the variable “PM2.5 air pollution, population exposed to levels exceeding WHO guideline value” estimates the population exposed to the PM2.5 levels exceeding specified threshold of 10 μg/m³.

The choice of covariates in our study was informed by previous literature [55,56]. The covariates in our study included energy use, FDI (% of GDP), forest cover, fossil fuel (%), CO2 emissions, GDP per capita, population density, urban population (%), electricity production, and GDP (energy use) (Table 1).

We employ panel data estimation techniques to empirically assess the impact of plastic bag bans and taxation policies on environmental quality, focusing on PM2.5 levels. We apply three estimation methods. First, Fixed Effects (FE) Model, which controls for unobserved, time-invariant characteristics of each country that may correlate with the explanatory variables. By using within-country variation over time, the FE model mitigates omitted variable bias due to unobserved heterogeneity [57]. This method is suitable when the unobserved effect is correlated with the explanatory variables. Second, Driscoll-Kraay (DK) method extends the FE model by providing standard errors that are robust to heteroskedasticity, autocorrelation, and cross-sectional dependence [58]. This is particularly important in panel data with large cross-sectional dimensions, where standard FE estimates may be inefficient or biased due to these issues. The Generalized Method of Moments (GMM) estimator, developed by Blundell and Bond [59], addresses potential endogeneity and measurement errors in the regressors by using lagged levels and differences of the variables as instruments. GMM is effective for dynamic panel data models where the dependent variable may be influenced by its own past values, and where some explanatory variables are endogenous. It also handles unobserved country-specific effects and serial correlation [60].

Data for the environmental variables were obtained from the World Bank's World Development Indicators and WHO databases [54], and data for the ban and tax regimes in individual countries were available at Wikipedia [61]. However, PM2.5 observations were only available for specific years (1990, 1995, 2000, 2005, 2010–2017), resulting in missing values for other years. To address this, we employed interpolation methods to estimate the missing PM2.5 values. Natural Cubic Spline Interpolation is a smooth interpolation method that fits a cubic polynomial between data points, ensuring continuity in the first and second derivatives [62,63]. The Piecewise Cubic Hermite Interpolation (PCHIP) preserves the shape and monotonicity of the data, avoiding overshooting and providing more accurate estimates for datasets with sharp changes [64,65]. These interpolation methods are well-regarded for handling missing data in environmental and economic studies [66,67]. This approach's effectiveness is demonstrated in Table 2, where the mean and standard deviation of variables remain consistent post-interpolation.

**Table 2:** Summary statistics.

Variable	N	Mean	SD	Min	Max	Range	CV^*(a)*^	Skewness	Kurtosis
**Original variables**									
PM2.5 (mcg)	2256	28.1	17.5	5.9	100.8	94.9	0.6	1.5	5.6
PM2.5 (% exposed) T-1	2256	24.7	38.2	0.0	100.0	100.0	1.6	1.2	2.7
PM2.5 (% exposed) T-2	2256	45.8	44.2	0.0	100.0	100.0	1.0	0.2	1.2
PM2.5 (% exposed) T-3	2256	74.3	38.5	0.0	100.0	100.0	0.5	−1.1	2.5
PM2.5 (% exposed)	2256	92.5	22.8	0.0	100.0	100.0	0.2	−3.2	11.9
**Interpolated variables**^***(b)***^									
PM2.5 (mcg)	11777	28.7	17.3	5.9	100.8	94.9	0.6	1.5	5.3
PM2.5 (% exposed) T-1	11642	25.9	38.6	0.0	100.0	100.0	1.5	1.1	2.5
PM2.5 (% exposed) T-2	11619	47.9	43.9	0.0	100.0	100.0	0.9	0.1	1.2
PM2.5 (% exposed) T-3	11601	76.5	37.2	0.0	100.0	100.0	0.5	−1.3	2.8
PM2.5 (% exposed)	11607	93.3	21.4	0.0	100.0	100.0	0.2	−3.4	13.2
**Covariates**									
Energy use	3801	5.6	3.8	0.1	32.6	32.5	0.7	2.4	10.4
FDI (% of GDP)	8181	5.5	45.6	−1303.1	1709.8	3012.9	8.3	15.8	567.9
Forest cover	6213	32.8	25.0	0.0	98.6	98.6	0.8	0.5	2.4
Fossil fuel (%)	5729	66.0	30.7	0.0	100.0	100.0	0.5	−0.7	2.2
CO2 emissions	5557	4.4	5.5	0.0	47.7	47.7	1.3	2.7	13.7
GDP per capita	5714	17780.5	19859.9	436.4	120647.8	120211.4	1.1	1.9	6.9
Population density	12140	231.6	1119.7	0.1	18288.6	18288.5	4.8	11.8	156.5
Urban population (%)	12710	51.0	25.3	2.1	100.0	97.9	0.5	0.1	2.0
Electricity production	5876	59.1	34.4	0.0	100.0	100.0	0.6	−0.4	1.7
GDP (energy use)	3303	9.6	7.7	1.0	224.7	223.7	0.8	15.2	383.5

(a) Coefficient of variation.

(b) Natural cubic spline and piecewise cubic Hermite interpolations were used to fill in the missing values. In case of variables about the population exposed to PM2.5, the interpolated values smaller than 0 and larger than 100 (*N* < 200) were again converted to missing values.

Despite the precautions taken, any interpolated values falling below 0 or above 100 (with a sample size less than 200) were converted back to missing values in the variables related to population exposed to PM2.5. This step was necessary to avoid any potential distortion of the dataset's underlying structure.

A review of the summary statistics given in Table 2, comparing raw and interpolated variables, demonstrates the preservation of the original data structure. Nonetheless, it is important to acknowledge the potential limitations associated with this approach. Although interpolation techniques have proven useful in these contexts, they remain estimations and, as such, might not completely represent the true values that would have been observed. This inherent limitation may slightly affect the precision of our results, which should be considered when interpreting the study's findings.

## 4 Results

Table 3 gives the pairwise correlation matrix. All five outcome variables are significantly correlated with ban at 10 % significance level, whereas the correlation with tax is insignificant in case of PM2.5 (mcg) and PM2.5 (% exposed) T-1.

**Table 3:** Pairwise correlations.

	Variables	(1)	(2)	(3)	(4)	(5)	(6)	(7)
(1)	PM2.5 (mcg)	1.00						
(2)	PM2.5 (% exposed) T-1	0.86∗	1.00					
(3)	PM2.5 (% exposed) T-2	0.79∗	0.75∗	1.00				
(4)	PM2.5 (% exposed) T-3	0.61∗	0.43∗	0.67∗	1.00			
(5)	PM2.5 (% exposed)	0.36∗	0.21∗	0.34∗	0.60∗	1.00		
(6)	Ban	0.18∗	0.21∗	0.17∗	0.11∗	0.05∗	1.00	
(7)	Tax	−0.04	−0.04	−0.05∗	−0.06∗	−0.08∗	0.00	1.00

Note: Asterisk indicates significance level at less than 10 %.

### 4.1 Ban on the use of plastic bags

**Fig. 1:**
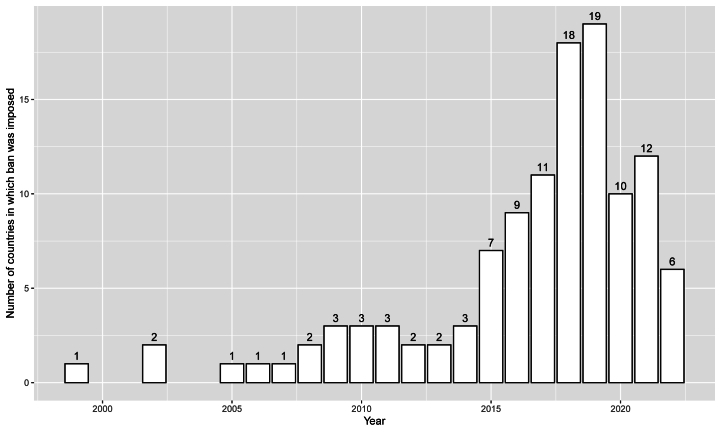
Ban on the use of plastic bags.

Source: Wikipedia [61].

Fig. 1 gives the number of countries which banned the use of plastic bags in a given year. There has been an overall increasing trend in the number of countries imposing bans on plastic bags. This pattern aligns with increasing global awareness regarding plastic pollution and efforts to mitigate it. The year 2015 appears to be a turning point with a notable increase in the number of countries banning plastic bags. The years following 2015 show a rapid increase, with 2018 and 2019 seeing the most countries implement a ban. Notably, the data shows a slight decrease in the rate of new bans from 2020 onwards. This could be due to a variety of reasons, such as the majority of countries already having imposed bans, political shifts, or potential distractions from other global events such as the COVID-19 pandemic.

### 4.2 Tax on the use of plastic bags

**Fig. 2:**
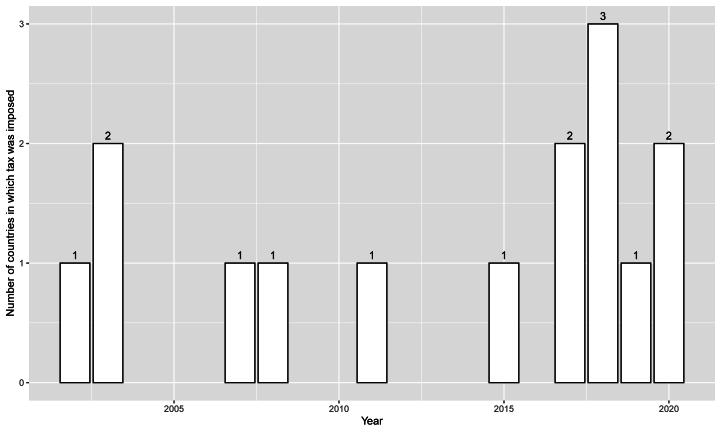
Tax on the use of plastic bags.

Source: Wikipedia [61].

Regarding the tax on plastic bags, we do not see a clear pattern because tax has been imposed in only 15 countries so far (Fig. 2).

### 4.3 Regression analysis

#### 4.3.1 Ban

Table 4 shows the unadjusted models in which the Ban significantly reduces PM2.5 exposure in various models: mean exposure (model 1, *β* = −1.39, *p* *<* 0.001), population share above T1 (model 2, *β* = −1.08, *p* *<* 0.01), T2 (model 3, *β* = −7.53, *p* *<* 0.001), and T3 (model 4, *β* = −7.25, *p* *<* 0.001). It also decreases population share (model 5, *β* = −4.10, *p* *<* 0.001). However, the GMM model shows the Ban increases mean exposure (model 6, *β* = 0.48, *p* *<* 0.001) and population share above T1 (model 7, *β* = 0.41, *p* *<* 0.001), but reduces share above T2 (model 8, *β* = −1.60, *p* *<* 0.001) and T3 (model 9, *β* = −0.48, *p* *<* 0.001), and increases population share (model 10, *β* = 0.18, *p* *<* 0.001).

**Table 4:** Ban (unadjusted model).

PM2.5 (a)		Mean	T1	T2	T3	Pop (%)
Ban	Fixed effects	(1)	(2)	(3)	(4)	(5)
		−1.39∗∗∗	−1.08∗∗∗	−7.53∗∗∗	−7.25∗∗∗	−4.10∗∗∗
		(-17.05)	(-5.76)	(-20.86)	(-24.04)	(-18.07)
Ban	Driscoll and Kraay	(6)	(7)	(8)	(9)	(10)
		−1.39∗∗∗	−1.08∗∗	−7.53∗∗∗	−7.25∗∗∗	−4.10∗∗∗
		(-3.36)	(-2.63)	(-4.84)	(-5.55)	(-5.45)
Ban	GMM	(11)	(12)	(13)	(14)	(15)
		0.48∗∗∗	0.41∗∗∗	−1.60∗∗∗	−0.48∗∗∗	0.18∗∗∗
		(109.70)	(2328.66)	(-5821.55)	(-1256.32)	(543.07)

*t* statistics in parentheses.

∗*p* < 0.05, ∗∗*p* < 0.01, ∗∗∗*p* < 0.001.

(a) The outcome variables Mean, T1, T2, T3, and Pop (%) denote mean annual exposure to PM2.5 (μg/m³) in a given area, population share exposed to PM2.5 air pollution levels exceeding WHO Interim Target-1 value, Target-2 value, Target-3 value, and population share exposed to PM2.5 air pollution levels exceeding WHO guideline value, respectively.

In the adjusted model (Table 5), the Ban notably reduces population exposure to PM2.5 surpassing WHO guidelines (model 5, *β* = −1.00, *p* *<* 0.05). The Driscoll and Kraay model shows an increase in exposure to PM2.5 above Interim Target-1 (model 7, *β* = 0.98, *p* *<* 0.05), but a decrease above Interim Target-3 and WHO guideline values (models 9 and 10, *β* = −1.13 and −1.00, *p* *<* 0.01 and < 0.05, respectively). The GMM model reveals mixed effects: increases in mean exposure (model 11, *β* = 0.49, *p* *<* 0.001) and share above Interim Target-3 (model 14, *β* = 1.16, *p* *<* 0.001), but decreases in exposure above Interim Target-1, Target-2, and WHO guidelines (models 12, 13, and 15, *β* = −0.91, −1.75, and −0.20, *p* *<* 0.001 and < 0.01).

**Table 5:** Ban (adjusted model).

PM2.5 ^(^a^*,*^^b^^)^		Mean	T1	T2	T3	Pop (%)
Ban	Fixed effects	(1)	(2)	(3)	(4)	(5)
		0.21	0.98	−0.84	−1.13	−1.00∗
		(0.79)	(1.62)	(-1.20)	(-1.56)	(-2.01)
	Driscoll and Kraay	(6)	(7)	(8)	(9)	(10)
		0.21	0.98∗	−0.84	−1.13∗∗	−1.00∗
		(0.37)	(2.08)	(-1.11)	(-3.35)	(-2.14)
	GMM	(11)	(12)	(13)	(14)	(15)
		0.49∗∗∗	−0.91∗∗∗	−1.75∗∗∗	1.16∗∗∗	−0.20∗∗
		(14.18)	(-29.76)	(-76.56)	(95.90)	(-2.69)

*t* statistics in parentheses.

∗*p* < 0.05, ∗∗*p* < 0.01, ∗∗∗*p* < 0.001.

(a) The column labels including Mean, T1, T2, T3, and Pop (%) are described in the note below Table 4.

(b) Additional covariates were estimated in the regression analysis for comprehensive assessment, including Energy Use, FDI (% of GDP), Forest Cover, Fossil Fuel (%), CO2 Emissions, GDP per Capita, Population Density, Urban Population (%), Electricity Production, GDP (Energy Use), and Lagged Values of PM2.5 (mcg) and PM2.5 Exposure Percentages (T-1, T-2, T-3) up to four periods. These have been omitted in the table for brevity.

#### 4.3.2 Tax

Table 6: The Tax leads to PM2.5 exposure reductions in the fixed effects model: mean exposure (model 1, *β* = −2.17, *p* *<* 0.001), population share above T1, T2, T3 (models 2–4, *β* = −1.95, −8.73, −4.84, all *p* *<* 0.001), and overall population share (model 5, *β* = −11.93, *p* *<* 0.001). The Driscoll and Kraay model confirms these effects. However, the GMM model shows Tax increases mean exposure (model 6, *β* = 0.48, *p* *<* 0.001) and population share above T1 and T2 (models 7 and 8, *β* = 0.26 and 0.21, *p* *<* 0.001), but decreases share above T3 (model 9, *β* = −1.25, *p* *<* 0.001) and overall population share (model 10, *β* = −2.16, *p* *<* 0.001).

**Table 6:** Tax (unadjusted model).

PM2.5		Mean	T1	T2	T3	Pop (%)
Tax	Fixed effects	(1)	(2)	(3)	(4)	(5)
		−2.17∗∗∗	−1.95∗∗∗	−8.73∗∗∗	−4.84∗∗∗	−11.93∗∗∗
		(-11.57)	(-4.55)	(-10.44)	(-6.79)	(-23.16)
Tax	Driscoll and Kraay	(6)	(7)	(8)	(9)	(10)
		−2.17∗	−1.95∗	−8.73∗∗∗	−4.84∗∗∗	−11.93∗∗∗
		(-2.55)	(-2.08)	(-3.78)	(-3.49)	(-5.61)
Tax	GMM	(11)	(12)	(13)	(14)	(15)
		0.48∗∗∗	0.26∗∗∗	0.21∗∗∗	−1.25∗∗∗	−2.16∗∗∗
		(15.30)	(86.31)	(86.67)	(-286.86)	(-2108.21)

*t* statistics in parentheses.

∗*p* < 0.05, ∗∗*p* < 0.01, ∗∗∗*p* < 0.001.

In the adjusted model (Table 7), Tax increases population exposure to PM2.5 above WHO Interim Target-3 (model 4, *β* = 2.93, *p* *<* 0.01). The Driscoll and Kraay model shows increased mean exposure (model 6, *β* = 0.68, *p* *<* 0.01) and population share above Interim Target-1 (model 7, *β* = 1.10, *p* *<* 0.01) and Target-3 (model 9, *β* = 2.93, *p* *<* 0.05). The GMM model indicates comprehensive increases in mean exposure (model 11, *β* = 0.47, *p* *<* 0.001) and population share above all examined levels (models 12–15, *β* = 0.33, 1.48, 3.21, 1.55, all *p* *<* 0.001).

**Table 7:** Tax (adjusted model).

PM2.5 ^(^a^*,*^^b^^)^		Mean	T1	T2	T3	Pop (%)
Tax	Fixed effects	(1)	(2)	(3)	(4)	(5)
		0.68	1.10	1.31	2.93∗∗	−1.10
		(1.66)	(1.21)	(1.23)	(2.66)	(-1.45)
Tax	Driscoll and Kraay	(6)	(7)	(8)	(9)	(10)
		0.68∗∗	1.10∗∗	1.31	2.93∗	−1.10
		(2.75)	(3.17)	(1.29)	(2.07)	(-1.27)
Tax	GMM	(11)	(12)	(13)	(14)	(15)
		0.47∗∗∗	0.33∗∗∗	1.48∗∗∗	3.21∗∗∗	1.55∗∗∗
		(12.59)	(6.32)	(27.72)	(10.78)	(17.35)

*t* statistics in parentheses.

∗*p* < 0.05, ∗∗*p* < 0.01, ∗∗∗*p* < 0.001.

(a) The column labels including Mean, T1, T2, T3, and Pop (%) are described in the note below Table 4.

(b) The details of the covariates estimated in the regression model are given in the note of Table 5.

#### 4.3.3 Ban and tax

In Table 8 (Unadjusted Model), both Ban and Tax show significant effects on PM2.5 exposure in fixed effects models. Ban reduces mean exposure (model 1, *β* = −1.36, *p* *<* 0.001) and population share above T1, T2, T3 (models 2–5, *β* = −1.05, −7.42, −7.19, −3.95, *p* *<* 0.01 and < 0.001). Tax similarly reduces PM2.5 measures. The Driscoll and Kraay models confirm these effects. The GMM models show Ban increases mean exposure (model 6, *β* = 0.52, *p* *<* 0.001) and population share above T1 (model 7, *β* = 0.41, *p* *<* 0.001), while Tax decreases these measures and positively impacts population share above T2 (model 8, *β* = 1.05, *p* *<* 0.001), but negatively impacts T3 (model 9, *β* = −0.66, *p* *<* 0.001) and overall population share (model 10, *β* = −2.52, *p* *<* 0.001).

**Table 8:** Ban and tax (unadjusted model).

PM2.5 (a)		Mean	T1	T2	T3	Pop (%)
		(1)	(2)	(3)	(4)	(5)
Ban	Fixed effects	−1.36∗∗∗	−1.05∗∗∗	−7.42∗∗∗	−7.19∗∗∗	−3.95∗∗∗
		(-16.79)	(-5.63)	(-20.64)	(-23.88)	(-17.77)
Tax		−2.07∗∗∗	−1.87∗∗∗	−8.21∗∗∗	−4.33∗∗∗	−11.66∗∗∗
		(-11.19)	(-4.38)	(-9.99)	(-6.22)	(-22.92)
		(6)	(7)	(8)	(9)	(10)
Ban	Driscoll and Kraay	−1.36∗∗∗	−1.05∗∗	−7.42∗∗∗	−7.19∗∗∗	−3.95∗∗∗
		(-3.42)	(-2.65)	(-4.92)	(-5.63)	(-5.73)
Tax		−2.07∗∗	−1.87∗	−8.21∗∗∗	−4.33∗∗∗	−11.66∗∗∗
		(-2.62)	(-2.11)	(-3.98)	(-3.98)	(-5.84)
		(11)	(12)	(13)	(14)	(15)
Ban	GMM	0.52∗∗∗	0.41∗∗∗	−1.65∗∗∗	−0.44∗∗∗	0.38∗∗∗
		(101.26)	(1820.48)	(-2015.58)	(-2177.29)	(479.02)
Tax		−0.42∗∗∗	0.09∗∗∗	1.05∗∗∗	−0.66∗∗∗	−2.52∗∗∗
		(-26.63)	(8.31)	(101.49)	(-116.98)	(-654.97)

*t* statistics in parentheses.

∗*p* < 0.05, ∗∗*p* < 0.01, ∗∗∗*p* < 0.001.

(a) The column labels including Mean, T1, T2, T3, and Pop (%) are described in the note below Table 4.

In the adjusted model (Table 9), the Ban reduces exposure to PM2.5 above WHO guidelines (model 5, *β* = −1.04, *p* *<* 0.05), while Tax increases exposure above Interim Target-3 (model 4, *β* = 2.85, *p* *<* 0.01). The Driscoll and Kraay model shows the Ban increases exposure above Interim Target-1 (model 7, *β* = 1.02, *p* *<* 0.05) but decreases higher levels (models 9 and 10). Tax increases mean annual exposure (model 6, *β* = 0.70, *p* *<* 0.05) and levels above Interim Target-1 (model 7, *β* = 1.17, *p* *<* 0.01). The GMM model indicates the Ban and Tax affect all outcome variables differently.

**Table 9:** Ban and tax (adjusted model).

PM2.5 ^(^a^*,*^^b^^)^		Mean	T1	T2	T3	Pop (%)
		(1)	(2)	(3)	(4)	(5)
Ban	Fixed effects	0.24	1.02	−0.80	−1.04	−1.04∗
		(0.87)	(1.68)	(-1.14)	(-1.44)	(-2.09)
Tax		0.70	1.17	1.25	2.85∗∗	−1.17
		(1.70)	(1.29)	(1.17)	(2.59)	(-1.55)
		(6)	(7)	(8)	(9)	(10)
Ban	Driscoll and Kraay	0.24	1.02∗	−0.80	−1.04∗∗∗	−1.04∗
		(0.40)	(2.14)	(-1.04)	(-3.47)	(-2.14)
Tax		0.70∗	1.17∗∗	1.25	2.85∗	−1.17
		(2.58)	(3.24)	(1.18)	(2.06)	(-1.34)
		(11)	(12)	(13)	(14)	(15)
Ban	GMM	0.49∗∗∗	−0.87∗∗∗	−1.70∗∗∗	1.04∗∗∗	−0.26∗∗∗
		(17.43)	(-35.50)	(-46.17)	(36.16)	(-5.02)
Tax		0.37∗∗∗	0.37∗∗∗	1.46∗∗∗	2.87∗∗∗	1.20∗∗∗
		(12.58)	(11.12)	(39.70)	(8.19)	(6.92)

*t* statistics in parentheses.

∗*p* < 0.05, ∗∗*p* < 0.01, ∗∗∗*p* < 0.001.

(a) The column labels including Mean, T1, T2, T3, and Pop (%) are described in the note below Table 4.

(b) The details of the covariates estimated in the regression model are given in the note of Table 5.

## 5 Discussion

The unmodified models typically indicate that both taxes and bans are effective in reducing PM2.5 exposure. Among these, the fixed effects model demonstrates the most significant impact compared to the other two methods. However, when the model was refined by including a range of covariates, the findings become more complex. The revised model still supports the notion that banning plastic bags markedly lowers PM2.5 exposure, yet, paradoxically, it also seems to increase the risk of such exposure. This counterintuitive shift in the impact of taxes suggests that certain practices, which might influence PM2.5 exposure, were not captured in the simpler models. As we further refine these models, the situation becomes increasingly intricate. The Generalized Method of Moments (GMM) in particular reveals a more complicated scenario, where even the plastic bag ban emerges as a potential risk factor for PM2.5 exposure under certain conditions.

Previous research supports the efficacy of both taxation and bans on single-use plastic bags in reducing their consumption and mitigating associated environmental pollutants [68,69]. Consistent with our unmodified models, studies by Convery, McDonnell [43] and Thomas, Sautkina [69] have demonstrated significant declines in plastic bag usage following the implementation of taxes and charges, highlighting the potential of these policies to decrease environmental contaminants. However, our refined models reveal a more complex interplay, echoing the findings of Muposhi, Mpinganjira [70], who identified that while plastic bag bans can effectively reduce certain types of pollution, they may inadvertently lead to increased use of alternative materials that do not necessarily lower PM2.5 levels. Additionally, Chien, Sadiq [71] emphasize that environmental taxes, when not comprehensively designed, can have unintended consequences on air quality, aligning with our observation that taxes might paradoxically elevate PM2.5 exposure under specific conditions. Furthermore, the nuanced outcomes uncovered through the Generalized Method of Moments (GMM) in our study resonate with Adam, Walker [72], who noted that insufficient policy frameworks and enforcement can undermine the intended environmental benefits of plastic bag regulations.

### 5.1 Ban on the plastic bags decrease air pollution

Public policy plays a crucial role in mitigating environmental problems, a role that has recently gained increasing recognition. A prime example of this fundamental role was the significant reduction in pollution levels during the COVID-19 pandemic, induced by global lockdowns. Studies in Poland, India, and China observed that these lockdowns resulted in a notable decrease in PM 2.5 levels, demonstrating the pivotal importance of public policy in addressing environmental challenges [[73], [74], [75], [76], [77]].

A key aspect of public policy in addressing environmental challenges involves imposing bans on products considered hazardous to the environment, such as plastic bags. Policymakers argue that reducing the use of hazardous materials improves environmental quality. Decreasing plastic bag usage reduces plastic waste and pollution, thereby lowering air pollution levels due to fewer emissions from disposal and processing. The production of plastic bags, involving the extraction and processing of crude oil and natural gas, significantly contributes to pollution [38,78].

The ban on plastic bags also indirectly affects environmental quality by promoting alternative, sustainable shopping bags. These reusable bags, often made from biodegradable and compostable materials, have gained popularity. A shift from plastic bags to these sustainable alternatives can potentially yield long-term air quality improvements. Where bans are in place, consumer behavior has markedly shifted toward reusable bag alternatives. Such behavioral changes, if sustained, can become social norms, leading to significant long-term environmental quality improvements. Moreover, improved plastic waste management, a critical aspect of policies targeting plastic usage, requires significant energy, which impacts environmental quality. Less plastic production and reduced waste management energy requirements can thus lead to a decrease in PM 2.5 levels [[79], [80], [81], [82]].

The effectiveness of public policies in reducing air pollution due to plastic bag bans also depends on implementation strategies and public perceptions of these policies. For a ban to be effective, it must be rigorously implemented, with the public viewing it not just as a legal obligation but as a moral imperative. The availability of affordable, alternative bags is also crucial to the ban's success. Easy access to affordable alternatives likely increases compliance, thereby reducing pollution [83].

### 5.2 Ban on the plastic bags increases air pollution

While the positive effects of plastic bag bans have been well researched, it is crucial to recognize that bans may also have unintended consequences, potentially increasing plastic bag usage. This paradox can arise from shifts in consumer behavior; when consumers switch from plastic bags to alternatives, those alternatives may have their environmental liabilities, potentially exacerbating pollution. This shift may increase reliance on materials used in alternative bags, which could be as harmful as, if not more than, traditional plastic bags [38,84].

Not all alternative bags are inherently environmentally friendly. For example, polypropylene nonwoven bags (PNBs) are environmentally beneficial only if used multiple times. Other alternatives, like cotton and Kraft paper bags, may have their environmental drawbacks, such as abiotic fossil depletion and acidification [85]. Inadequate or improper disposal and waste management of these alternative bags can also lead to environmental issues, including litter, landfill accumulation, and the release of microplastics or other pollutants. Moreover, the extraction, processing, and transportation of raw materials for alternative bag manufacturing can negatively impact the environment and increase air pollutants. A comprehensive life-cycle analysis and assessment of the environmental impact of alternative bags are vital to ensure their environmental effects are less severe than those of plastic bags [78,86,87].

A ban on plastic bags could also stimulate increased production of other plastic products. For instance, when a plastic bag ban was implemented in some regions of China, air pollution increased as plastic production shifted to other sectors. The ban might also lead to increased consumption of other single-use items. Evidence suggests that when consumers are prohibited from using plastic bags, they may turn to other non-prohibited bags with equally adverse environmental impacts [84,88,89].

Finally, the effectiveness of a ban significantly depends on its implementation, rigorous enforcement, and comprehensive public education. Literature highlights the importance of effective policy promotion, stakeholder engagement, and awareness campaigns. Strict, comprehensive enforcement and monitoring are crucial to achieving clean air objectives. However, the effectiveness of bans can be undermined if suitable alternatives are unavailable or if there is limited public sector capacity to enforce the ban, due to scarce resources or corruption. Ineffective enforcement can lead to loopholes, such as the use of free inner plastic wrapping bags, which may have greater environmental impacts. Studies have shown that strict ban policies can expose loopholes in policy execution, potentially leading to greater plastic bag usage [83,[90], [91], [92], [93]].

While banning plastic bags is a strategy aimed at reducing air pollution, the resultant increase in the use of alternative materials, enforcement loopholes, and possible increases in the production of other plastic products may paradoxically exacerbate air pollution. Hence, comprehensive, context-specific strategies and rigorous enforcement mechanisms are needed to ensure that plastic bag bans achieve their intended environmental benefits [91].

### 5.3 Tax increase air pollution

This conflicting evidence about the effect of tax on the plastic bag consumption and by implication on the air quality points to the complexity of environmental policies and their varied impacts on consumer behavior and industry practices. In Ireland, a 15 Euro cent environmental levy implemented in 2002 on shopping plastic bags led to a significant 90 % reduction in their consumption, thereby positively affecting the landscape [94]. Portugal's experience in 2015 was similar, with a plastic bag tax resulting in a 74 % decrease in usage and a 61 % increase in reusable bag consumption, demonstrating the tax's effectiveness in fostering sustainable habits [95]. However, the implementation of plastic bag taxes, while aiming to mitigate their non-biodegradable impact, has in some cases paradoxically been linked to an increase in PM2.5 exposure, an outcome attributed to a mix of behavioral, economic, and industrial factors which are explained below.

Firstly, the behavioral response to plastic bag taxation is complex. While taxes aim to discourage the use of plastic bags, they may inadvertently lead to the increased use of alternative products with similar or worse environmental impacts. For example, consumers might switch to paper bags or non-reusable plastic alternatives, which can lead to higher emissions in their production and disposal processes. This shift undermines the anticipated environmental benefits of reducing plastic bag usage and contributes to increased air pollution, including higher PM2.5 levels [96].

Furthermore, the effectiveness of the tax in changing consumer behavior varies by region. In some areas, the plastic bag tax has not significantly altered consumer behavior. The persistence of plastic bag usage, particularly in regions like Islamabad, suggests the tax's ineffectiveness in reducing plastic consumption. This continued use of plastic bags, despite the tax, does not help in diminishing the associated pollution, thereby contributing to increased PM2.5 levels [38].

Additionally, the tax has influenced waste processing activities, especially in countries with substantial plastic waste imports. In China, for instance, the introduction of plastic bag taxes led to an increase in waste processing activities, particularly in recycling and incineration sectors. These increased activities have inadvertently contributed to higher PM2.5 emissions, directly correlating with the heightened waste processing efforts necessitated by the reduced, yet significant, volume of plastic waste post-taxation [38].

The results of this study suggest several policy implications that can be addressed through short-term, medium-term, and long-term strategies to manage PM2.5 exposure while mitigating plastic pollution. In the short term, enhancing the enforcement of existing plastic bag bans and taxes could ensure compliance and reduce the use of harmful alternatives. Additionally, public engagement and education campaigns may raise awareness about the environmental impacts of plastic bags and encourage responsible disposal practices. In the medium term, adopting a comprehensive policy design that considers the entire lifecycle of alternative products may prevent substitutes from exacerbating pollution, supported by lifecycle assessments to identify truly sustainable materials. Reevaluating taxation policies to address unintended consequences, such as potential increases in PM2.5 from alternative product usage, while ensuring equitable implementation could help mitigate economic impacts on lower-income households. In the long term, integrating plastic bag regulations with broader environmental strategies, including alignment with air quality regulations and investment in waste management and recycling infrastructure, may enhance overall environmental outcomes. Furthermore, transitioning to a circular economy by promoting reusable and recyclable product designs and implementing extended producer responsibility policies could sustain reductions in PM2.5 exposure. These phased, evidence-based approaches offer a framework for achieving effective and balanced environmental outcomes based on the nuanced relationships identified in our study.

### 5.4 Limitations

This study's dataset, encompassing 208 countries, offers a global scope but may not directly apply to individual countries due to unique socio-economic and political contexts. It identifies correlations between plastic bag policies and PM2.5 levels but falls short of establishing causality, potentially overlooking confounding factors. The assumption of static policy effects over time may not reflect the dynamic nature of societal adaptations, and the use of PM2.5 as an air quality proxy could introduce measurement inaccuracies. Additionally, the study does not fully consider variations in policy enforcement and implementation, nor does it explore the interaction effects between bans and taxes. Despite using fixed-effects models and GMM, unmeasured variables may still influence the outcomes. The study also omits analysis of mediating factors such as consumer behavior and industrial responses. Future research should focus on context-specific causal mechanisms, dynamic policy impacts, refined measurements, and the integration of interactional and mediating factors.

## 6 Conclusion

This study presents an insightful examination of the relationship between plastic bag policies and air pollution levels, providing critical evidence for policy deliberations on environmental protection. Our analysis reveals that the implementation of plastic bag bans generally reduces population exposure to PM2.5 pollution, whereas taxation increases exposure across several measures. However, these effects are not uniform across all WHO target levels, indicating intricate dynamics and potential trade-offs in policy impacts. The finding of increased PM2.5 exposure following the implementation of a tax on plastic bags particularly merits further investigation, as it suggests the possibility of unforeseen consequences of such policies.

## CRediT authorship contribution statement

**Rafi Amir-ud-Din:** Writing – review & editing, Supervision, Methodology, Formal analysis, Data curation, Conceptualization. **Muhammad Khan:** Resources, Project administration, Funding acquisition, Conceptualization. **Rao Muhammad Atif:** Writing – review & editing, Supervision, Resources, Project administration. **Saliha Khalid:** Writing – review & editing, Writing – original draft, Formal analysis, Conceptualization.

## Data availability statement

Data have been deposited at Mendeley Data with https://doi.org/10.17632/2mtjyr97xd.1.

## Institutional review

We used publicly available data, and hence no institutional review was required.

## Ethics declarations

Since the study utilised cross-country macro data from secondary sources and did not involve any human subjects, ethical approval was not required.

## Use of generative AI and AI-assisted technologies

The authors used ChatGPT to correct language and improve readability of the manuscript. The authors take full responsibility for all the contents of this study.

## Funding

This work was supported by the 10.13039/501100010221Higher Education Commission of Pakistan under Grand Challenge Fund vide GCF-860.

## Declaration of competing interest

The authors declare the following financial interests/personal relationships which may be considered as potential competing interests:Muhammad Khan reports financial support was provided by Higher Education Commission. If there are other authors, they declare that they have no known competing financial interests or personal relationships that could have appeared to influence the work reported in this paper.
