# Supplementary material for: The paradox of plastic bag legislation: How bans and taxes affect PM2.5 air pollution in 208 countries
Source: Heliyon. 2024 Nov 22;10(23):e40641. doi: 10.1016/j.heliyon.2024.e40641 (PMC11626041; doi:10.1016/j.heliyon.2024.e40641)
Supplement: Multimedia component 1 [file mmc1.docx]

# Online Supplement

Table A1: Ban and Tax

| Sr. | Country Name | Ban | Tax | Ban *or* Tax | Ban *and* Tax | Year | |
| --- | --- | --- | --- | --- | --- | --- | --- |
|  |  |  |  |  |  | Ban | Tax |
| 1 | Afghanistan | 1 | 0 | 1 | 0 | 2012 |  |
| 2 | Albania | 1 | 0 | 1 | 0 | 2018 |  |
| 3 | Algeria | 0 | 0 | 0 | 0 |  |  |
| 4 | American Samoa | 0 | 0 | 0 | 0 |  |  |
| 5 | Andorra | 1 | 0 | 1 | 0 | 2017 |  |
| 6 | Angola | 0 | 0 | 0 | 0 |  |  |
| 7 | Antigua and Barbuda | 0 | 0 | 0 | 0 |  |  |
| 8 | Argentina | 1 | 0 | 1 | 0 | 2017 |  |
| 9 | Armenia | 1 | 0 | 1 | 0 | 2022 |  |
| 10 | Aruba | 0 | 0 | 0 | 0 |  |  |
| 11 | Australia | 1 | 0 | 1 | 0 | 2018 |  |
| 12 | Austria | 1 | 0 | 1 | 0 | 2020 |  |
| 13 | Azerbaijan | 1 | 0 | 1 | 0 | 2021 |  |
| 14 | Bahamas, The | 1 | 0 | 1 | 0 | 2020 |  |
| 15 | Bahrain | 1 | 0 | 1 | 0 | 2019 |  |
| 16 | Bangladesh | 1 | 0 | 1 | 0 | 2002 |  |
| 17 | Barbados | 1 | 0 | 1 | 0 | 2019 |  |
| 18 | Belarus | 0 | 0 | 0 | 0 |  |  |
| 19 | Belgium | 0 | 0 | 0 | 0 |  |  |
| 20 | Belize | 1 | 0 | 1 | 0 | 2019 |  |
| 21 | Benin | 1 | 0 | 1 | 0 | 2017 |  |
| 22 | Bermuda | 0 | 0 | 0 | 0 |  |  |
| 23 | Bhutan | 1 | 0 | 1 | 0 | 1999 |  |
| 24 | Bolivia | 0 | 0 | 0 | 0 |  |  |
| 25 | Bosnia and Herzegovina | 0 | 0 | 0 | 0 |  |  |
| 26 | Botswana | 1 | 1 | 1 | 1 | 2018 | 2007 |
| 27 | Brazil | 1 | 0 | 1 | 0 | 2009 |  |
| 28 | British Virgin Islands | 0 | 0 | 0 | 0 |  |  |
| 29 | Brunei Darussalam | 0 | 0 | 0 | 0 |  |  |
| 30 | Bulgaria | 0 | 1 | 1 | 0 |  | 2011 |
| 31 | Burkina Faso | 1 | 0 | 1 | 0 | 2015 |  |
| 32 | Burundi | 1 | 0 | 1 | 0 | 2020 |  |
| 33 | Cambodia | 1 | 0 | 1 | 0 | 2017 |  |
| 34 | Cameroon | 1 | 0 | 1 | 0 | 2014 |  |
| 35 | Canada | 1 | 0 | 1 | 0 | 2022 |  |
| 36 | Cayman Islands | 0 | 0 | 0 | 0 |  |  |
| 37 | Central African Republic | 0 | 0 | 0 | 0 |  |  |
| 38 | Chad | 1 | 0 | 1 | 0 | 2010 |  |
| 39 | Channel Islands | 0 | 0 | 0 | 0 |  |  |
| 40 | Chile | 1 | 0 | 1 | 0 | 2015 |  |
| 41 | China | 0 | 1 | 1 | 0 |  | 2008 |
| 42 | Colombia | 1 | 0 | 1 | 0 | 2017 |  |
| 43 | Comoros | 1 | 0 | 1 | 0 | 2018 |  |
| 44 | Congo, Dem Rep | 1 | 0 | 1 | 0 | 2018 |  |
| 45 | Congo, Dem. Rep. | 1 | 0 | 1 | 0 | 2022 |  |
| 46 | Congo, Rep | 0 | 0 | 0 | 0 |  |  |
| 47 | Costa Rica | 1 | 0 | 1 | 0 | 2021 |  |
| 48 | Cote d'Ivoire | 0 | 0 | 0 | 0 |  |  |
| 49 | Croatia | 1 | 0 | 1 | 0 | 2022 |  |
| 50 | Cuba | 0 | 0 | 0 | 0 |  |  |
| 51 | Curacao | 0 | 0 | 0 | 0 |  |  |
| 52 | Cyprus | 0 | 1 | 1 | 0 |  | 2018 |
| 53 | Czech Republic | 0 | 0 | 0 | 0 |  |  |
| 54 | Denmark | 0 | 1 | 1 | 0 |  | 2003 |
| 55 | Djibouti | 1 | 0 | 1 | 0 | 2016 |  |
| 56 | Dominica | 1 | 0 | 1 | 0 | 2019 |  |
| 57 | Dominican Republic | 0 | 0 | 0 | 0 |  |  |
| 58 | Ecuador | 1 | 0 | 1 | 0 | 2020 |  |
| 59 | Egypt, Arab Rep | 0 | 0 | 0 | 0 |  |  |
| 60 | Egypt, Arab Rep. | 1 | 0 | 1 | 0 | 2022 |  |
| 61 | El Salvador | 0 | 0 | 0 | 0 |  |  |
| 62 | Equatorial Guinea | 0 | 0 | 0 | 0 |  |  |
| 63 | Eritrea | 1 | 0 | 1 | 0 | 2006 |  |
| 64 | Estonia | 1 | 0 | 1 | 0 | 2017 |  |
| 65 | Ethiopia | 1 | 0 | 1 | 0 | 2008 |  |
| 66 | Faroe Islands | 0 | 0 | 0 | 0 |  |  |
| 67 | Fiji | 1 | 0 | 1 | 0 | 2020 |  |
| 68 | Finland | 0 | 0 | 0 | 0 |  |  |
| 69 | France | 1 | 0 | 1 | 0 | 2016 |  |
| 70 | French Polynesia | 0 | 0 | 0 | 0 |  |  |
| 71 | Gabon | 1 | 0 | 1 | 0 | 2010 |  |
| 72 | Gambia, The | 1 | 0 | 1 | 0 | 2015 |  |
| 73 | Georgia | 1 | 0 | 1 | 0 | 2018 |  |
| 74 | Germany | 1 | 0 | 1 | 0 | 2021 |  |
| 75 | Ghana | 0 | 0 | 0 | 0 |  |  |
| 76 | Gibraltar | 0 | 0 | 0 | 0 |  |  |
| 77 | Greece | 1 | 0 | 1 | 0 | 2018 |  |
| 78 | Greenland | 0 | 0 | 0 | 0 |  |  |
| 79 | Grenada | 1 | 0 | 1 | 0 | 2019 |  |
| 80 | Guam | 0 | 0 | 0 | 0 |  |  |
| 81 | Guatemala | 1 | 0 | 1 | 0 | 2021 |  |
| 82 | Guinea | 0 | 0 | 0 | 0 |  |  |
| 83 | Guinea-Bissau | 1 | 0 | 1 | 0 | 2016 |  |
| 84 | Guyana | 1 | 0 | 1 | 0 | 2021 |  |
| 85 | Haiti | 0 | 0 | 0 | 0 |  |  |
| 86 | Honduras | 1 | 0 | 1 | 0 | 2019 |  |
| 87 | Hungary | 1 | 0 | 1 | 0 | 2012 |  |
| 88 | Iceland | 1 | 0 | 1 | 0 | 2021 |  |
| 89 | India | 1 | 0 | 1 | 0 | 2002 |  |
| 90 | Indonesia | 1 | 0 | 1 | 0 | 2019 |  |
| 91 | Iran, Islamic Rep | 0 | 0 | 0 | 0 |  |  |
| 92 | Iraq | 0 | 0 | 0 | 0 |  |  |
| 93 | Ireland | 0 | 1 | 1 | 0 |  | 2002 |
| 94 | Israel | 1 | 0 | 1 | 0 | 2017 |  |
| 95 | Italy | 1 | 0 | 1 | 0 | 2011 |  |
| 96 | Jamaica | 1 | 0 | 1 | 0 | 2019 |  |
| 97 | Japan | 1 | 0 | 1 | 0 | 2020 |  |
| 98 | Jordan | 0 | 0 | 0 | 0 |  |  |
| 99 | Kazakhstan | 0 | 0 | 0 | 0 |  |  |
| 100 | Kenya | 1 | 0 | 1 | 0 | 2017 |  |
| 101 | Kiribati | 1 | 0 | 1 | 0 | 2020 |  |
| 102 | Korea, Rep | 0 | 0 | 0 | 0 |  |  |
| 103 | Kuwait | 0 | 0 | 0 | 0 |  |  |
| 104 | Kyrgyz Republic | 1 | 0 | 1 | 0 | 2018 |  |
| 105 | Lao PDR | 0 | 0 | 0 | 0 |  |  |
| 106 | Latvia | 1 | 0 | 1 | 0 | 2019 |  |
| 107 | Lebanon | 1 | 0 | 1 | 0 | 2018 |  |
| 108 | Lesotho | 0 | 0 | 0 | 0 |  |  |
| 109 | Liberia | 0 | 0 | 0 | 0 |  |  |
| 110 | Libya | 0 | 0 | 0 | 0 |  |  |
| 111 | Liechtenstein | 0 | 0 | 0 | 0 |  |  |
| 112 | Lithuania | 1 | 0 | 1 | 0 | 2018 |  |
| 113 | Luxembourg | 0 | 0 | 0 | 0 |  |  |
| 114 | Madagascar | 1 | 0 | 1 | 0 | 2015 |  |
| 115 | Malawi | 1 | 0 | 1 | 0 | 2015 |  |
| 116 | Malaysia | 1 | 0 | 1 | 0 | 2011 |  |
| 117 | Maldives | 1 | 0 | 1 | 0 | 2021 |  |
| 118 | Mali | 1 | 0 | 1 | 0 | 2013 |  |
| 119 | Malta | 1 | 0 | 1 | 0 | 2022 |  |
| 120 | Marshall Islands | 0 | 0 | 0 | 0 |  |  |
| 121 | Mauritania | 1 | 0 | 1 | 0 | 2013 |  |
| 122 | Mauritius | 1 | 0 | 1 | 0 | 2016 |  |
| 123 | Mexico | 1 | 0 | 1 | 0 | 2009 |  |
| 124 | Micronesia, Fed Sts | 1 | 0 | 1 | 0 | 2020 |  |
| 125 | Moldova | 1 | 0 | 1 | 0 | 2021 |  |
| 126 | Monaco | 1 | 0 | 1 | 0 | 2016 |  |
| 127 | Mongolia | 1 | 0 | 1 | 0 | 2019 |  |
| 128 | Montenegro | 0 | 0 | 0 | 0 |  |  |
| 129 | Morocco | 1 | 0 | 1 | 0 | 2016 |  |
| 130 | Mozambique | 1 | 0 | 1 | 0 | 2016 |  |
| 131 | Myanmar | 1 | 0 | 1 | 0 | 2009 |  |
| 132 | Namibia | 1 | 0 | 1 | 0 | 2018 |  |
| 133 | Nauru | 1 | 0 | 1 | 0 | 2021 |  |
| 134 | Nepal | 0 | 0 | 0 | 0 |  |  |
| 135 | Netherlands | 1 | 0 | 1 | 0 | 2016 |  |
| 136 | New Caledonia | 0 | 0 | 0 | 0 |  |  |
| 137 | New Zealand | 1 | 0 | 1 | 0 | 2018 |  |
| 138 | Nicaragua | 0 | 0 | 0 | 0 |  |  |
| 139 | Niger | 0 | 0 | 0 | 0 |  |  |
| 140 | Nigeria | 1 | 0 | 1 | 0 | 2014 |  |
| 141 | North Korea | 1 | 0 | 1 | 0 | 2018 |  |
| 142 | Norway | 1 | 0 | 1 | 0 | 2020 |  |
| 143 | Oman | 1 | 0 | 1 | 0 | 2021 |  |
| 144 | Pakistan | 1 | 0 | 1 | 0 | 2019 |  |
| 145 | Palau | 1 | 0 | 1 | 0 | 2019 |  |
| 146 | Panama | 1 | 0 | 1 | 0 | 2019 |  |
| 147 | Papua New Guinea | 1 | 0 | 1 | 0 | 2016 |  |
| 148 | Paraguay | 0 | 0 | 0 | 0 |  |  |
| 149 | Peru | 1 | 0 | 1 | 0 | 2019 |  |
| 150 | Philippines | 0 | 0 | 0 | 0 |  |  |
| 151 | Poland | 1 | 0 | 1 | 0 | 2018 |  |
| 152 | Portugal | 0 | 1 | 1 | 0 |  | 2017 |
| 153 | Puerto Rico | 0 | 0 | 0 | 0 |  |  |
| 154 | Qatar | 0 | 0 | 0 | 0 |  |  |
| 155 | Romania | 1 | 0 | 1 | 0 | 2018 |  |
| 156 | Russian Federation | 0 | 0 | 0 | 0 |  |  |
| 157 | Rwanda | 1 | 0 | 1 | 0 | 2008 |  |
| 158 | Samoa | 0 | 0 | 0 | 0 |  |  |
| 159 | San Marino | 1 | 0 | 1 | 0 | 2021 |  |
| 160 | Sao Tome and Principe | 0 | 0 | 0 | 0 |  |  |
| 161 | Saudi Arabia | 0 | 0 | 0 | 0 |  |  |
| 162 | Senegal | 1 | 0 | 1 | 0 | 2015 |  |
| 163 | Serbia | 0 | 1 | 1 | 0 |  | 2018 |
| 164 | Seychelles | 1 | 0 | 1 | 0 | 2017 |  |
| 165 | Sierra Leone | 0 | 0 | 0 | 0 |  |  |
| 166 | Singapore | 0 | 0 | 0 | 0 |  |  |
| 167 | Slovak Republic | 0 | 1 | 1 | 0 |  | 2017 |
| 168 | Slovenia | 0 | 1 | 1 | 0 |  | 2019 |
| 169 | Solomon Islands | 0 | 0 | 0 | 0 |  |  |
| 170 | Somalia | 1 | 0 | 1 | 0 | 2005 |  |
| 171 | South Africa | 0 | 1 | 1 | 0 |  | 2003 |
| 172 | Spain | 0 | 1 | 1 | 0 |  | 2018 |
| 173 | Sri Lanka | 1 | 0 | 1 | 0 | 2017 |  |
| 174 | St Kitts and Nevis | 0 | 0 | 0 | 0 |  |  |
| 175 | St Lucia | 0 | 0 | 0 | 0 |  |  |
| 176 | St Vincent and the Grenadines | 0 | 0 | 0 | 0 |  |  |
| 177 | Sudan | 1 | 0 | 1 | 0 | 2015 |  |
| 178 | Suriname | 0 | 0 | 0 | 0 |  |  |
| 179 | Sweden | 0 | 1 | 1 | 0 |  | 2020 |
| 180 | Switzerland | 0 | 1 | 1 | 0 |  | 2020 |
| 181 | Syrian Arab Republic | 0 | 0 | 0 | 0 |  |  |
| 182 | Tajikistan | 0 | 0 | 0 | 0 |  |  |
| 183 | Tanzania | 1 | 0 | 1 | 0 | 2019 |  |
| 184 | Thailand | 1 | 0 | 1 | 0 | 2020 |  |
| 185 | Timor-Leste | 0 | 0 | 0 | 0 |  |  |
| 186 | Togo | 1 | 0 | 1 | 0 | 2018 |  |
| 187 | Tonga | 0 | 0 | 0 | 0 |  |  |
| 188 | Trinidad and Tobago | 0 | 0 | 0 | 0 |  |  |
| 189 | Tunisia | 1 | 0 | 1 | 0 | 2017 |  |
| 190 | Turkey | 1 | 0 | 1 | 0 | 2019 |  |
| 191 | Turkmenistan | 0 | 0 | 0 | 0 |  |  |
| 192 | Turks and Caicos Islands | 0 | 0 | 0 | 0 |  |  |
| 193 | Tuvalu | 1 | 0 | 1 | 0 | 2019 |  |
| 194 | Uganda | 1 | 0 | 1 | 0 | 2007 |  |
| 195 | Ukraine | 1 | 0 | 1 | 0 | 2021 |  |
| 196 | United Arab Emirates | 0 | 0 | 0 | 0 |  |  |
| 197 | United Kingdom | 1 | 1 | 1 | 1 | 2018 | 2015 |
| 198 | United States | 1 | 0 | 1 | 0 | 2014 |  |
| 199 | Uruguay | 1 | 0 | 1 | 0 | 2019 |  |
| 200 | Uzbekistan | 1 | 0 | 1 | 0 | 2019 |  |
| 201 | Vanuatu | 1 | 0 | 1 | 0 | 2018 |  |
| 202 | Venezuela, RB | 0 | 0 | 0 | 0 |  |  |
| 203 | Vietnam | 0 | 0 | 0 | 0 |  |  |
| 204 | Virgin Islands (US) | 0 | 0 | 0 | 0 |  |  |
| 205 | West Bank and Gaza | 0 | 0 | 0 | 0 |  |  |
| 206 | Yemen, Rep | 0 | 0 | 0 | 0 |  |  |
| 207 | Zambia | 1 | 0 | 1 | 0 | 2011 |  |
| 208 | Zimbabwe | 1 | 0 | 1 | 0 | 2010 |  |
|  | **Total** | **116** | **15** | **129** | **2** |  |  |

Source: [Wikipedia](https://en.wikipedia.org/wiki/Plastic_bag_ban)
